# Different Gain/Loss Sensitivity and Social Adaptation Ability in Gifted Adolescents during a Public Goods Game

**DOI:** 10.1371/journal.pone.0017044

**Published:** 2011-02-16

**Authors:** Dongil Chung, Kyongsik Yun, Jin Ho Kim, Bosun Jang, Jaeseung Jeong

**Affiliations:** 1 Department of Bio and Brain Engineering, Korea Advanced Institute of Science and Technology (KAIST), Daejeon, Republic of Korea; 2 Division of Electrical Engineering, Korea Advanced Institute of Science and Technology (KAIST), Daejeon, Republic of Korea; 3 Department of Physics, Korea Advanced Institute of Science and Technology (KAIST), Daejeon, Republic of Korea; University of Maribor, Slovenia

## Abstract

Gifted adolescents are considered to have high IQs with advanced mathematical and logical performances, but are often thought to suffer from social isolation or emotional mal-adaptation to the social group. The underlying mechanisms that cause stereotypic portrayals of gifted adolescents are not well known. We aimed to investigate behavioral performance of gifted adolescents during social decision-making tasks to assess their affective and social/non-social cognitive abilities. We examined cooperation behaviors of 22 gifted and 26 average adolescents during an iterative binary public goods (PG) game, a multi-player social interaction game, and analyzed strategic decision processes that include cooperation and free-riding. We found that the gifted adolescents were more cooperative than average adolescents. Particularly, comparing the strategies for the PG game between the two groups, gifted adolescents were less sensitive to loss, yet were more sensitive to gain. Additionally, the behavioral characteristics of average adolescents, such as low trust of the group and herding behavior, were not found in gifted adolescents. These results imply that gifted adolescents have a high cognitive ability but a low ability to process affective information or to adapt in social groups compared with average adolescents. We conclude that gain/loss sensitivity and the ability to adapt in social groups develop to different degrees in average and gifted adolescents.

## Introduction

Gifted adolescents are generally considered to have higher IQs and better mathematical and logical performances than average age-matched individuals. Intellectually gifted adolescents show superior performance (e.g., better memory, faster and more efficient processing) on various cognitive tasks, including visuo-spatial tasks (e.g., 3-dimensional mental rotation) that require creativity and manipulation of mental images [1], [2], problem solving [3], [4], memory processing [5], [6], and global-local processing [7]. In contrast to their advanced performances in cognitive tasks, however, they are often thought to have low sensitivity to social cues within their age group, or to be socially isolated [8]. Although the stereotyped view of maladjustment is controversial in other studies [9]–[11], little is known about the reasons why gifted adolescents are often thought to show maladaptive behaviors in their group, especially in environments in which social interactions are required.

Social decision-making requires complex information processing, including the integration of cognitive and affective information and the prediction of others' future behavior [12]. Thus, matured cognitive and affective processing are essential for strategic decision-making in order to maximize profit he or she can earn from the group. Gifted adolescents are often judged to be emotionally maladapted to social groups [13], [14]. Thus, gifted adolescents can be expected to show superior performance in cognitive tasks, but poor performance in integrative tasks that include affective information. Investigating choices to cooperate or not with a participating group is an apt tool for evaluating the factors, including self-maximizing (to maximize one's monetary gain) and emotional reactions (e.g., avoiding the specific option more than other option irrationally such as loss aversion effect), that motivate a participant in strategic decision-making. In reacting to information provided during such a task, participants who depend less on emotional information processing should show more (monetary) gain-sensitive behavior than (monetary) loss-sensitive behavior (i.e., projection of emotional reaction). Since a participant should weigh affective and cognitive motivations before making decisions, critical decision differences can be expected between gifted and average adolescents. However, potential behavioral and neuro-developmental differences between gifted and average adolescents during social interactions have rarely been investigated.

The aim of the current study was first to investigate behavioral strategies for cooperation and free-riding in mathematically gifted adolescents with high IQs during a public goods (PG) game, a multiple-player social interaction game; and second, to analyze behaviors to discern the major causes of behavioral differences between gifted and average adolescents if exists. We hypothesized that gifted adolescents would show gain-sensitive strategies, that maximize their mean total earnings with the superior mathematical abilities that help them to react adaptively to any given environment. Gifted adolescents were also expected to be less affected by emotional factors (loss-insensitive) than average adolescents. These different characteristics of strategic and social decision-making might cause maladaptations to social groups in daily life.

A PG game is a useful tool for assessing social interactions, particularly through cooperative and free-riding behaviors. Participants are given a certain amount of money at the beginning of the game and should decide whether to invest their money in the public account or in a private account (i.e., to keep it). Public money is generally doubled or tripled and is shared equally between players regardless of their cooperation. According to game theory, the dominant strategy to earn the most is to free-ride, in which a player selfishly keeps his or her own money and also earns a group share, while investment of the entire money from all participants in the public account generates the Pareto-efficient outcome – sharing the most efficient and fair amount of payoff between the assigned group members [15]–[18]. However, in empirical studies, participants showed around 20–40% cooperation (i.e., invested in the public account) in one-shot PG games or in the first round of repeated PG games [15]–[22]. Thus, emergence of non-kin cooperation has been broadly investigated on the point of view of evolutionary game theory and suggested that reciprocity, group selection [23], [24], and coevolutionary rule (e.g., environmental evolution) [25] are the possible mechanisms for the evolution of cooperation. Furthermore, it has been shown that cooperative behaviors are promoted by social diversity [26] and institutional designs that implement punishment or reward [27]–[31]. Besides modulating the incentives, however, according to the strategic decisions of the participants, initial cooperation quickly diminished and converged to nearly 0% cooperation in later rounds of repeated PG games [15]–[18], [32].

We used an iterative binary PG game, a well-controlled design for evaluating the behavioral performance of the gifted and average adolescents during strategic social decision-making. First, a binary design of a PG game, based on Dawes et al. [19], was chosen for simplicity. This game provides only two options for decisions, i.e., cooperate or free-ride, and two alternative results, i.e., success and failure to earn a bonus. An amount of twice the promissory note was provided if and only if more than 3 of 5 participants cooperated. Second, three differentiated conditions were applied in order to distinguish the two major incentives to free-ride, which are ‘fear of losing money’ and ‘greed for earning more money than others’ [19]. The first condition (condition I) included the standard social dilemma problem in the game. Two other conditions (condition II and III) had the two ‘half social dilemma problems’ [19], including each of the incentive to free-ride, respectively. We analyzed the possible behavioral differences that were induced by each of the modified incentives and compared gifted with average adolescents in their strategic social decision-making abilities. Third, we used a 10-round PG game. Iterative decisions in response to the given information and individual gain or loss results reflect individual risk-aversive or risk-taking characteristics. Our results uncover underlying differences in the decision-making processes of gifted and average adolescents that account for social maladaptation and isolation problems.

## Materials and Methods

### Ethics Statement

Before participating in the experiment, all recruited students and their teachers were informed about all procedures of the experiment and written informed consents were obtained. All protocols utilized in the current study were reviewed and approved by the KAIST institutional review board (KH2008-01).

### Subjects

Twenty four gifted adolescents (age range: 13–15 years, M:F = 18∶6) and forty average adolescents (age range: 13–14 years, M:F = 24∶16) were initially recruited for the current study. For the gifted adolescent group, students from a private institute for special education for the gifted were recruited. In order to obtain a large pool of the gifted, the gifted adolescents were recruited independently from the average, through local private academy. All the gifted participants had received awards from local or national competition which qualifying their advanced mathematical and logical performances. A teacher additionally participated in the game to make each group consist of five non-overlapping players, but data from the teacher was not included in the data analysis. After recruiting, we tried to make groups of minimal acquaintance by asking who knows whom and avoiding them to be in the same group. For the average group, we recruited first-year middle-school students from the Gapcheon middle school, Daejeon, South Korea, with the assistance of teachers in the school. As a control group to be compared with gifted adolescents, students were randomly recruited regardless of their academic records. To minimize the influence of participants' acquaintance with each other, we took average applications from 4 students each from 10 different classes.

All participants in both groups took an IQ test, the Wechsler Intelligence Scale for Children, Third Edition [33], and a creativity test, the Khatena-Torrance Creative Perception Inventory (KTCPI [34]), prior to other procedures. IQ was measured to divide the two groups with the objective threshold and to rule out the subjects who have incongruent traits (i.e., adolescent from the gifted population who has low IQ, or from the average population who has high IQ). The threshold for the IQ separation was set to 130. Creativity is another measure that often refers to criteria of the giftedness [35]. Since the correlation between creativity and intelligence is controversial (negligible vs. modestly related in part) [36]–[38], the current study measured KTCPI score and focused on effects of the sub-components which showed positive correlation with IQ.

We excluded 3 gifted adolescents who had IQs lower than 130, and 14 average adolescents who had IQs equal to or higher than 130, from analyses to make a further distinction between the gifted and average groups. The PG game provided a well-preserved anonymity for individual decisions, and the only information given to the players was the result of the group in each round (i.e., success or failure to earn a bonus) and the supportiveness of the group (i.e., the number of cooperators in the preceding trial). Hence, although the excluded students participated in the game, we assumed that each participant made his or her decisions independently and that only the given information affected their decisions. According to the exclusion criteria, we finally analyzed data from 26 average adolescents (age: 13.96±0.20 years, age range: 13–14 years, M/F: 15/11) and 22 gifted adolescents (age: 14.05±0.49 years, age range: 13–15 years, M/F: 16/6).

### Experimental Procedures

The experimental procedures were carried out independently for the gifted and the average groups. We utilized the binary PG game based on Dawes et al. [19], which provides only two choices for the participant: to cooperate, to invest all money in the public; or to free-ride, to keep all of the money to one's self. Five randomly-selected participants were grouped as a team. In each round, players were provided promissory notes of 1,000 Korean won (about 1 US dollar), and decided to invest the money in the public or their private account. Their cooperation determined the result of the group, i.e., whether everyone earned a bonus or not. A bonus of twice the first provided endowment, i.e., $2, was given to all participants regardless of his or her decision only if 3 or more of 5 participants cooperated during the trial. Otherwise, the publicly invested money was not paid back. These procedures were repeated for 10 rounds to investigate strategic behavior during the game.

Additionally, we provided two more conditions with modified incentives. In the second condition (condition II), participants were provided exactly the same environment as the standard game design (condition I) except that we offered the guarantee that the participants who invested money into the public would be paid back if the group failed to satisfy the bonus threshold. The third condition (condition III) had a different incentive modification from condition II. The participants were not guaranteed their money back as in condition I. Instead, they were enforced to cooperate for a bonus that was unequally provided according to participants' decisions. The bonus was adjusted to make fair net earnings, i.e., $2, in the corresponding successful trial. When the group succeeded, participants who cooperated were given $2, twice as much as the amount they invested, whereas the free-riders were provided with $1. Since the free-riders did not invest their money (initial promissory note; $1), the extra one dollar for the free-riders make the net earnings as $2 (equal to the cooperators' net earnings). We examined these three differentiated conditions to investigate how average and gifted adolescents performed in each condition, and to reveal their strategic processes during social interactions, which required the integration of affective and cognitive information and the interpretation of others' actions.

Eight teams of average adolescents and five teams of gifted adolescents participated in the current study. They were instructed before the game not to communicate with each other, and communication was also prevented by the wearing of masks distributed during the instruction. Written protocols for the PG game were provided individually and were also orally explained to each group. A simple questionnaire, including four examples of each of the three conditions, was given to ensure the students understood all of the possible cases that could occur during the game. They were instructed to sit around the table facing each other and to repeat 10 rounds of the PG game for each condition (i.e., condition I, II and III). We used two types of cards as imaginary money for the game, with written numbers that represented the value of the card: 1,000 and 0 corresponding to $1 and $0, respectively. Participants were told that they would receive a gift certificate proportional to the amount of money they acquired after all three conditions of the game. All participants were provided with one ‘1,000’ card and one ‘0’ card for each round, and after some time for decision-making, they were each instructed to turn in one of the cards to the instructor simultaneously. They had to hand in the card face-down, to preserve anonymity of cooperation. After the decision, the instructor recorded the cooperation of each player, let the participants know whether the group could earn a bonus or not, and identified the number of cooperators in the preceding round. The instructor announced the remaining amount of trials at the beginning of each round.

### Data analysis

We measured cooperation rates, success or failure of the group in earning a bonus, and total earnings of the participants to analyze and compare performances. To investigate sequential effects of the players' decisions, we calculated cooperation rates and stay rates for each sub-case, grouped according to success or failure result, or the number of cooperators in the preceding trial. For each group, performances among three conditions were compared using the Kruskal-Wallis one-way analysis of variation. We used the Mann-Whitney U test for post-hoc tests. The stay-or-shift strategy was examined through one-sample Wilcoxon signed rank tests examining whether the participant's stay ratio was biased in either direction from a 50% chance of staying. The Spearman's correlation analysis was used to examine correlations between the participants' performances and their demographic characteristics. The alpha level was set to 0.05 for all statistical tests. The commercial statistical package SPSS 13.0 for windows (SPSS 13.0; SPSS Inc., Chicago, IL, USA) was used for all statistical analyses.

## Results

According to the exclusion criteria, we finally analyzed data from 26 average adolescents (age: 13.96±0.20 years, age range: 13–14 years, M/F: 15/11) and 22 gifted adolescents (age: 14.05±0.49 years, age range: 13–15 years, M/F: 16/6). The average group was not different from the gifted group in terms of age (χ^2^(1) = 0.657, p = 0.417) or sex (χ^2^(1) = 1.178, p = 0.278), but the average and gifted adolescents were statistically distinguishable in terms of IQ (χ^2^(1) = 35.066, p<0.001). Among KTCPI scores, the gifted group had significantly higher scores on the section ‘what kind of person are you’ (WKOPAY; χ^2^(1) = 3.917, p<0.05), whereas the average and gifted group had comparable scores on the section ‘something about myself’ (SAM; χ^2^(1) = 0.021, p = 0.885). Furthermore, the two groups were statistically different in regards to inquisitiveness (χ^2^(1) = 4.505, p<0.05) and disciplined imagination (χ^2^(1) = 7.734, p<0.01) sub-items of the WKOPAY scores. Demographic data of average and gifted adolescents are summarized in Table 1. The sub-items of KTCPI scores are described and summarized in Table 2. These demographic data of the two groups show that the current study had a well-controlled set of participant groups, particularly the gifted adolescents with distinguishable indexes.

**Table 1:** Demographic characteristics of average and gifted adolescents.

		Average (n = 26)		Gifted (n = 22)		
Variables		Mean	SD	Mean	SD	Significance level
Age (years)		13.96	0.20	14.05	0.49	χ^2^(1) = 0.657
						p = 0.417
Sex		15/11		16/6		χ^2^(1) = 1.178
(male/female)						p = 0.278
IQ*		111.11	13.01	142.59	5.95	χ^2^(1) = 35.066
						p<0.001
aKTCPI		57.90	18.38	66.09	23.06	χ^2^(1) = 1.952
						p = 0.162
bWKOPAY*		50.65	25.69	66.68	27.14	χ^2^(1) = 3.917
						p<0.05
cSAM		65.15	29.33	65.50	30.93	χ^2^(1) = 0.021
						p = 0.885

a KTCPI, Khatena-Torrance Creative Perception Inventory score;

b WKOPAY, What kind of person are you score;

c SAM, Something about myself score;

*, statistically significant differences between groups.

**Table 2:** Sub-item statistics of the KTCPI scores for average and gifted adolescents.

	Average (n = 26)	Gifted (n = 22)			
Variables	Mean	SD	Mean	SD	Significance level
aWKOPAY*	50.65	25.69	66.68	27.14	χ^2^(1) = 3.917
					p<0.05
Acceptance of authority	55.96	24.79	48.91	29.00	χ^2^(1) = 0.882
					p = 0.348
Self Confidence	55.00	32.29	58.41	26.84	χ^2^(1) = 0.118
					p = 0.732
Inquisitivness*	70.08	26.45	53.59	29.20	χ^2^(1) = 4.505
					p<0.05
Awareness of others	57.88	32.01	47.59	31.48	χ^2^(1) = 1.433
					p = 0.231
Disciplined Imagination*	65.88	27.12	83.64	21.11	χ^2^(1) = 7.734
					p<0.01
bSAM	65.15	29.33	65.50	30.93	χ^2^(1) = 0.021
					p = 0.885
Environmental sensitivity	48.81	33.28	53.23	37.18	χ^2^(1) = 0.001
					p = 0.975
Initiative(I)	65.65	24.80	65.18	27.74	χ^2^(1) = 0.007
					p = 0.933
Self-strength(SS)	70.42	27.48	68.91	25.89	χ^2^(1) = 0.013
					p = 0.909
Intellectuality	61.69	30.68	61.41	32.34	χ^2^(1) = 0.013
					p = 0.909
Individuality	51.88	30.77	66.91	25.13	χ^2^(1) = 1.986
					p = 0.159
Artistry(A)	56.15	33.31	45.82	32.46	χ^2^(1) = 0.843
					p = 0.358

a WKOPAY, What kind of person are you score;

b SAM, Something about myself score;

*, statistically significant differences between groups.

### Cooperation ratios in the PG game

**Figure 1:**
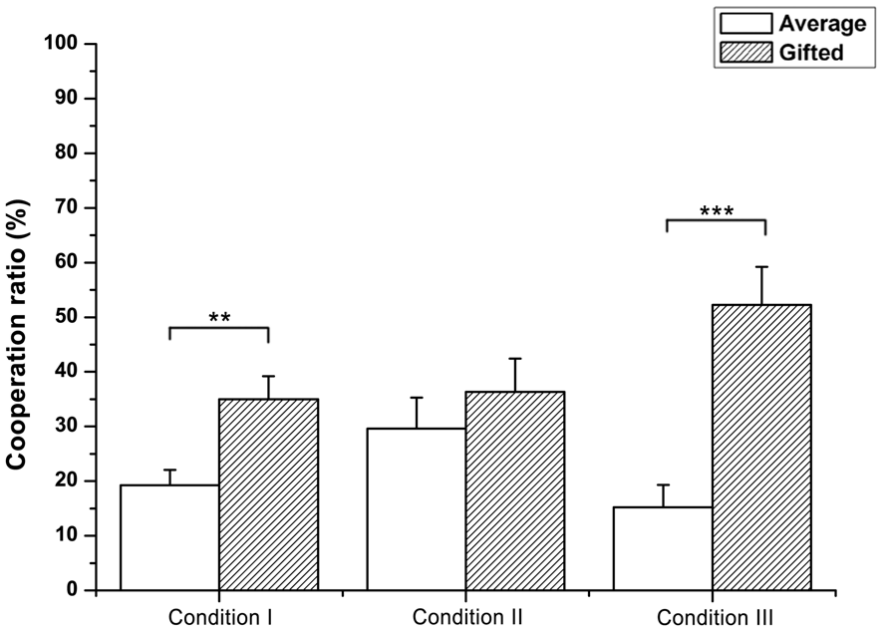
Mean cooperation ratios in each condition. Average and gifted adolescents showed statistically comparable cooperation ratios in all three conditions. The gifted group was significantly more cooperative in both conditions I and III, whereas the two groups had similar cooperation rates in condition II. Standard errors of each condition are represented as error bars; *p<0.05; **p<0.01; ***p<0.001

We first observed the cooperation behavior of the participants in each condition in the PG game. Through all three conditions, the adolescents showed rather low cooperation overall, ranging from around 15% to 50% at maximum (Fig. 1). Particularly in condition I, the gifted adolescents showed significantly higher cooperation (about 35%) than the average adolescents (about 20%) (χ^2^(1) = 8.994, p<0.01). In condition II, the gifted group still showed a higher mean cooperation rate (about 35%) than the average group (about 30%), but the difference was not significant. The gifted and average adolescents had the largest and most significant cooperation difference in condition III (χ^2^(1) = 14.901, p<0.001); the gifted group showed about 50% cooperation and the average group showed about 15% cooperation. This result indicates that the gifted adolescents were more cooperative than the average adolescents regardless of the condition design.

To examine whether each group was affected by incentive modifications, we compared the cooperation ratios between the conditions. Both groups did not show any significant differences in cooperation rates among the three conditions (gifted: χ^2^(2) = 3.844, p = 0.146; average: χ^2^(2) = 3.950, p = 0.139). However, we observed that the gifted adolescents tended to be more cooperative in condition III, in which cooperation was enforced, than in the other two conditions. These results demonstrate that greed possibly affects the gifted adolescents more than the average adolescents.

### Total earnings in the PG game

**Figure 2:**
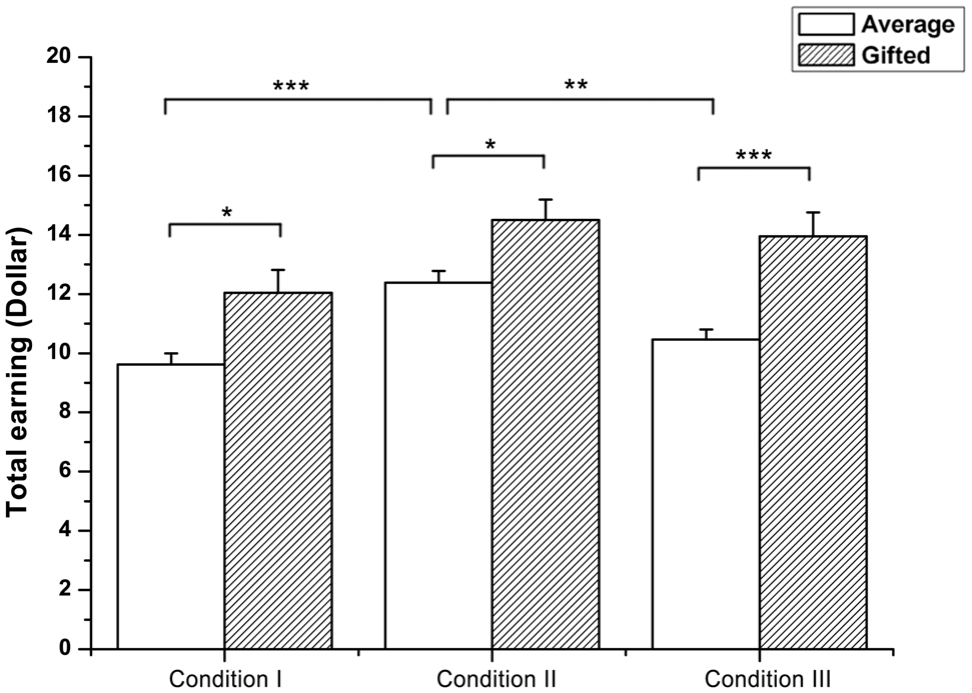
Mean total earnings in each condition. Gifted adolescents earned comparable amounts in three conditions. Average adolescents earned the most in condition II among the three conditions. The amount they earned in condition II was significantly higher than in conditions I and III. Compared with the average group, the gifted group earned significantly larger amounts in each condition. Black asterisk: within-group difference; Grey asterisk: between-group difference; Standard errors of each condition are represented as error bars; *p<0.05; **p<0.01; ***p<0.001

We found that the gifted adolescents earned more than the average adolescents in all three conditions (Fig. 2). In condition I, the gifted group earned about $12 and the average group earned about $10 on average; this difference was significant (χ^2^(1) = 5.951, p<0.05). In condition II, the gifted adolescents earned significantly more, about $14, than the average adolescents, about $12 (χ^2^(1) = 5.199, p<0.05). The gifted group also showed the superior performance to the average group in terms of mean total earnings in condition III, during which the gifted earned about $14 and the average earned about $12 (χ^2^(1) = 13.906, p<0.001). These results indicate that the gifted adolescents maximized their profit better than the average adolescents in most circumstance.

Comparing the monetary performances among the three conditions, the gifted group did not show any significant difference between different conditions (χ^2^(2) = 5.754, p = 0.056). However, the average group earned the most in condition II among the three conditions (χ^2^(2) = 21.822, p<0.001). Their mean total earnings in condition II were much higher than in condition I (U = 104.000, p<0.001) or condition III (U = 159.500, p<0.01). These results indicate that the average group found the condition that had no risk of losing much easier than other conditions when maximizing their profit. In other words, the average adolescents were sensitive to monetary loss during the game.

### Sequential effects on decisions in the PG game

**Figure 3:**
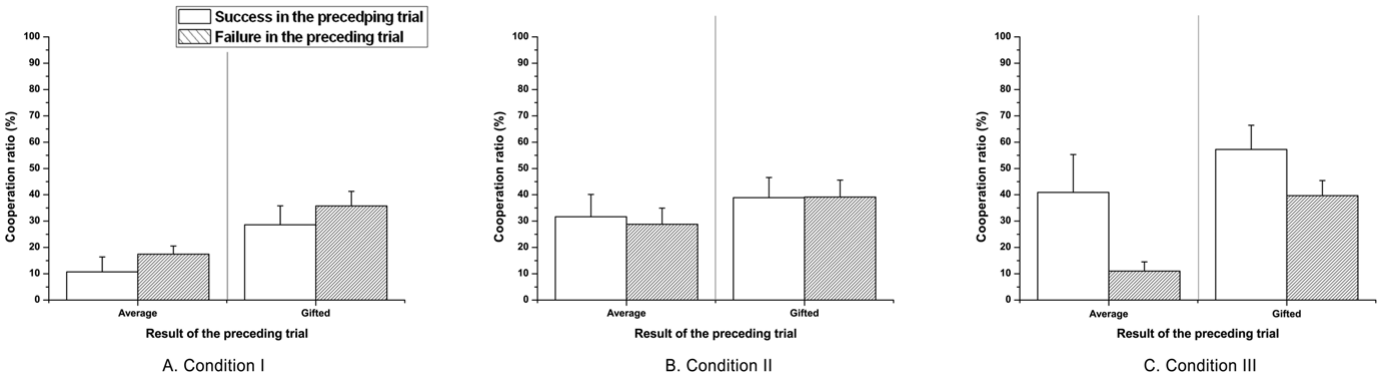
Mean cooperation ratios in a successive trial following the preceding successful or failed trials. Both groups had comparable cooperation rates regardless of the result of the preceding trial in (a) condition I, (b) condition II, and (c) condition III. Particularly in condition III, the average group exhibited relatively larger differences than the gifted group between successful and failed rounds.

To investigate strategic decision-making of adolescents in social interactions, we utilized a 10-round repeated design and estimated the sequential effects (Figure 1 in File S1). Particularly, we computed the cooperation ratios and stay-or-shift ratios in reorganized sub-cases that showed whether or not participants were affected by success or failure and by the number of cooperators in the preceding trial. The effect of the preceding trial's result was examined first. In conditions I, II and III, both groups showed no significant effects of success or failure in the preceding trial (Condition I: average, U = 117.500, p = 0.067; gifted, U = 192.000, p = 0.231; Condition II: average, U = 253.500, p = 0.881; gifted, U = 195.000, p = 0.934; Condition III: average, U = 99.000, p = 0.265; gifted, U = 112.000, p = 0.111; Fig. 3). These results show that there is no significant loss sensitivity difference between groups that revealed in terms of mean cooperation rates.

**Figure 4:**
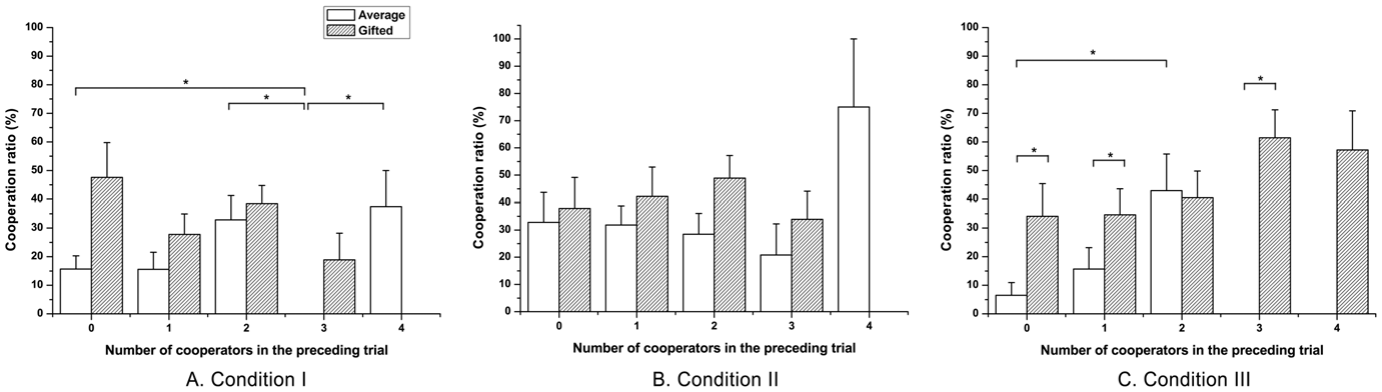
Mean cooperation ratios in a successful trial following trials with the indexed numbers of cooperators. (a) In condition I, gifted adolescents showed comparable cooperation through all sub-cases. In the gifted group, none of the cases had 4 preceding cooperators. Average adolescents exhibited significantly low cooperation following trials with 3 cooperators, as compared to those with 0, 2 and 4 cooperators. (b) In condition II, the gifted group had similar cooperation rates in all sub-cases except for the case with 4 cooperators in the preceding trial. The gifted group showed 0% cooperation rate in the corresponding case that is relatively lower than in trials with 0, 1 and 2 preceding cooperators. The average adolescents also showed comparable cooperation rates in all cases except for a relatively higher cooperation following 4 preceding cooperators. (c) In condition III, the gifted group had statistically comparable cooperation rates for all sub-cases. The average adolescents exhibited significantly higher cooperation in the trials, with 2 preceding cooperators when compared to those with 0 cooperators in condition III. None of the cases in the average group had 4 preceding cooperators. Between the two groups, the gifted adolescents showed significantly higher cooperation in which 0, 1 or 3 cooperators existed in the previous round. Black asterisk: within-group difference; Grey asterisk: between-group difference; Standard errors of each condition are represented as error bars; *p<0.05; **p<0.01; ***p<0.001

We recalculated the cooperation rate in each sub-case according to the number of cooperators in the preceding trial (0 to 4); this excludes the decisions of the corresponding participant. Under the assumption that two repeated, non-consecutive decisions were independent during the procedure, the effects of the given information on the behavioral change could be investigated (Figure 2 in File S1). Gifted adolescents showed comparable behaviors in all sub-cases during the condition I (χ^2^(3) = 6.475, p = 0.091), except that none of the cases had 4 cooperators in the preceding trial (Fig. 4A). Compared with the gifted group, average adolescents showed relatively diverse cooperation differences over the sub-cases (χ^2^(4) = 10.558, p<0.05). The average group exhibited 0% cooperation when there were 3 cooperators in the last round, in condition I. Interestingly, each of the cooperation rates following rounds with 0, 2 and 4 cooperators was significantly higher than the cooperation following trials in which 3 players cooperated (0: U = 42.000, p<0.05; 2: U = 24.000, p<0.05; 4: U = 3.000, p<0.05, respectively). The average group's low cooperation following trials in which the group had 3 cooperators might be accounted for by both fear and greed, while the relatively high cooperation following trials in which 4 participants cooperated shows an abnormal characteristic of average adolescents. These results indicate that the average adolescents are non-strategic compared to the gifted adolescents in the sense of maximizing the total earning.

In condition II, neither gifted nor average adolescents were affected significantly by the given information about the number of cooperators in the preceding trial (average: χ^2^(4) = 5.276, p = 0.260; gifted: χ^2^(4) = 4.660, p = 0.324). Although cooperation behaviors of the gifted group were not significantly different among the sub-cases, their 0% cooperation following the trials with 4 cooperators indicates relatively strong greed compared with the average adolescents (Fig. 4B).

In condition III, the gifted adolescents had significantly different cooperation ratios than the average adolescents in most of the sub-cases (Fig. 4C). The gifted group showed higher cooperation rates following trials in which they had 0, 1, and 3 cooperators (0: χ^2^(1) = 5.876, p<0.05; 1: χ^2^(1) = 3.935, p<0.05; 3: χ^2^(1) = 6.500, p<0.05, respectively). These results imply that the gifted adolescents are less sensitive to loss compared to the average adolescents.

Comparing cooperation within the group in condition III, the gifted adolescents did not change their cooperation significantly according to others' decisions in the preceding trial (χ^2^(4) = 5.488, p = 0.241). In contrast, the average adolescents showed significantly different cooperation rates between the sub-cases (χ^2^(3) = 9.003, p<0.05); in particular, they cooperated significantly more following trials in which 2 cooperated, compared with trials in which all participants free-rode (U = 86.500, p<0.05). Although the condition enforced cooperation, the average group was not cooperative. The results in the average group (i.e., 0% cooperation following 3 previous cooperators and no cases of 4 preceding cooperators) suggest that average adolescents' weak mind-reading abilities prevented them from reaching a collective decision as a group. On the contrary, the gifted participants' high cooperation following trials where more than 3 participants cooperated suggests that the gifted adolescents have better mind-reading abilities than average adolescents.

### Stay-or-shift strategic choices in the PG game

**Figure 5:**
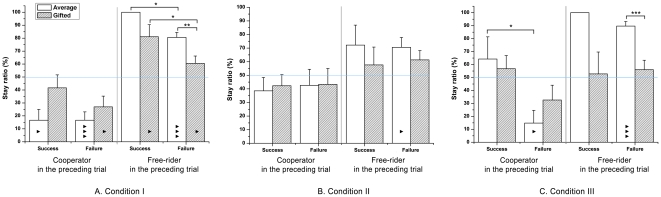
Mean strategic stay ratios in the trial following previously successful or failed trials. Each case of trials was tested for whether the ratios were significantly different from 50% chance of a changing strategy (horizontal blue line). (a) In condition I, the cooperators among the gifted adolescents shifted randomly after success, but shifted significantly after failure. The free-riders among the gifted adolescents showed a significant stay strategy following successful and failed trials. Among the average group, the cooperators always chose a shift strategy, whereas the free-riders always chose a stay strategy, regardless of the group result. The free-riders of the average group showed significantly more cooperation after success than after failure. (b) In condition II, cooperators and free-riders in both groups chose a random shift strategy in all possible cases except the following trials in which the free-riders among the average adolescents failed. They showed a significant stay strategy. (c) In condition III, the gifted group always shifted randomly from their corresponding alternative decision in all cases. In contrast, the cooperators among the average adolescents exhibited significant differences from the group result in that they randomly shifted after success and shifted after failure. The free-riders in the group chose a significant stay strategy regardless of the result. The free-riders of the average adolescents showed significantly higher stay ratios than the gifted adolescents, regardless of the group result. Black asterisk: within-group difference; Grey asterisk: between-group difference; Standard errors of each condition are represented as error bars; *p<0.05; **p<0.01; ***p<0.001; ^▴^p<0.05; ^▴▴^p<0.01; ^▴▴▴^p<0.001

We analyzed the participants' strategic drifts by calculating stay-or-shift ratios in each condition. Cooperators and free-riders were observed, and each case, followed by a successful or failed trial, was described (Fig. 5). By using one-sample Wilcoxon signed rank tests, we defined a stay ratio significantly higher than 50% as a ‘stay’ strategy, a stay ratio lower than 50% as a ‘shift’ strategy, and all other insignificant cases as ‘random shift’ (see Methods). The gifted adolescents showed more strategic drifts according to success or failure than the average adolescents in condition I (Fig. 5A). In the gifted group, a significant amount of cooperators shifted only if their group failed to earn a bonus (Z = −2.490, p<0.05) and free-riders stayed regardless of success or failure (Z = 2.495, p<0.05; Z = 1.969, p<0.05). However, the gifted adolescents shifted randomly when they cooperated and succeeded in earning a bonus. More interestingly, free-riders stayed significantly less when they failed as compared to the cases in which they succeeded (U = 87.500, p<0.05). These results indicate that the gifted adolescents are less affected by possible loss, but strongly chase the strategies that can maximize their profit (i.e., earning a bonus) or at least not losing their money.

On the other hand, in condition I, the average adolescents who cooperated in the preceding trial showed significant shifts to free-riding regardless of the result (success: Z = −2.333, p<0.05; failure: Z = −3.227, p<0.001). In contrast, the free-riders chose to stay in all circumstances (success: 100% stay; failure: Z = 4.186, p<0.001). In particular, the free-riders showed significantly higher stay ratios when the group succeeded (U = 22.500, p<0.05). These results suggest that the average adolescents prefer to be free-riders, due to either fear or greed.

In condition II, gifted adolescents chose to shift by random chance in all cases (Fig. 5B). As the condition guarantees money back in failed cases, the decisions of the gifted adolescents in condition II clearly followed rational processes, i.e., no stay-ratio differences between successful and failed trials. In contrast, among the average adolescents, the significant stay strategy that the free-riders used following trials in which they failed (Z = 2.407, p<0.05) implies a non-strategic behavior.

In condition III, the gifted adolescents shifted randomly regardless of success or failure or the choice they made (cooperate or free-ride) in the preceding trial (cooperator: U = 68.000, p = 0.120; free-rider: U = 79.000, p = 0.917). However, the average group exhibited loss-aversive behaviors that were not revealed by measuring cooperation ratios alone (Fig. 5C). The cooperators tended to shift after failed trials (Z = −2.373, p<0.05), whereas they did not choose a particular strategy following a success. The stay ratios between successful and failed cases were significantly different (U = 11.000, p<0.05). The free-riders always stayed regardless of the result of the preceding trial (success: 100% stay; failure: Z = 4.187, p<0.001), even though they had no chance of earning more than the cooperator, according to the rule of the condition. These results indicate that average adolescents have loss-sensitive behavior, but that gifted adolescents do not.

### Correlations between demographic data and performances

We additionally analyzed correlations between the participants' demographic data (i.e., IQ and KTCPI scores) and their performances in the game (i.e., cooperation rates, total earnings, and stay ratios). For simplicity, we only focused on the demographic data that were significantly different between the average and the gifted adolescents: IQ, average WKOPAY score, WKOPAY score in disciplined imagination, and inquisitiveness. The significant correlations we found are summarized in Table 1 in File S1.

**Figure 6:**
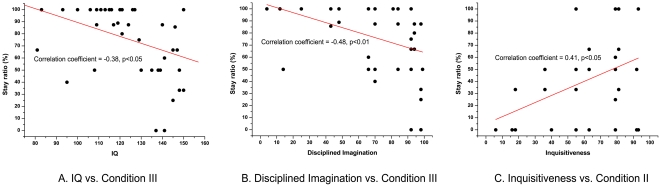
Significant correlations between demographic data and performances. A significant negative correlation was found between (a) IQs and stay ratios and (b) Disciplined imagination score and stay ratios of the free-riders when the group failed to earn a bonus in condition III, which indicates that participants with higher scores on the corresponding demographic data tended to shift at the corresponding case. (c) Stay ratios in condition II during which cooperators succeeded showed significant positive correlation with Inquisitiveness score, which represents that the gifted adolescents with low Inquisitiveness score tended to have higher greediness.

Among the significant correlations, we found clear clues supporting the notion that the gifted adolescents were strategically superior to the average adolescents. First, we found a negative correlation between IQs and stay ratios in condition III when free-riders failed in the preceding trial (Spearman's correlation coefficient = −0.38, p<0.05; Fig. 6A). The significant positive correlation was detected between IQs and the total earnings in the corresponding condition (Spearman's correlation coefficient = 0.43, p<0.01), which may result from the gifted adolescents' well-suited shift strategies. In condition III, we also found a significant negative correlation between the stay ratios of free-riders and disciplined imagination (Spearman's correlation coefficient = −0.48, p<0.01; Fig. 6B), one of the WKOPAY scores from the creativity test; this agrees with the correlation we mentioned above. Furthermore, we observed a positive correlation between stay ratios in condition II during which cooperators succeeded, and inquisitiveness, one of the WKOPAY scores from the creativity test (Spearman's correlation coefficient = 0.41, p<0.05; Fig. 6C). Based on this correlation observed (i.e., a participant with a higher inquisitiveness score tends to stay with the previous choice), we suggest that average adolescents, who showed significantly higher inquisitiveness than the gifted (Table 2), were not greedy. In other words, gifted adolescents tend to shift to free-riding, which gives them the opportunity to take advantage of others' cooperation.

## Discussion

The aim of the current study was to investigate the behavioral strategies of cooperation and free-riding in gifted adolescents during the PG game, and to analyze their behaviors to examine the major causes of behavioral differences between gifted and average adolescents. Between two groups, gifted adolescents were more cooperative, as observed in a previous study [39], and earned more than average adolescents. Gifted adolescents showed weak loss sensitivity but notable greed. Additionally, they were more strategic in that their behavior could be straightforwardly accounted for by economic, emotional or social motivation. In contrast, average adolescents were rather less cooperative than the gifted group, more sensitive to loss, non-greedy, and non-strategic in repeated decisions.

### Gain and loss sensitivity in the PG game

The current study found that gifted adolescents show more risk-taking behavior compared with average adolescents. First, gifted adolescents' diminished loss sensitivity seemed to cause their risk-taking behavior. In the PG game, particularly in condition III (greed-free environment), the participants might fear losing money due to the uncertain probability of earning a bonus. In decision theory, since the probability of earning a bonus remained uncertain through iterations, the participants' decisions should be random and not affected by others' choices [40]. However, in the empirical study, participants estimated the probability of winning based on the results of the preceding trials and became either risk-aversive or risk-taking. Thus, loss sensitivity may lead to emotion-based phenomena that fear of losing money causes differences in behavioral differences against risk [41]. Most interestingly, in strategic drifts of cooperators, the gifted group showed relatively lower and insignificant levels of loss sensitivity, compared with the average group. One possible causal brain mechanism that might underlie the behavioral differences is interactions between brain regions which encode cognitive and affective process. According to a neurobiological interpretation from a recent study, gifted adolescents might show more risk-taking behavior due to infrequent or weaker interaction between affective and cognitive control networks in the brain, as compared to average adolescents [42]. In contrast to gifted adolescents, average adolescents showed relatively stronger loss sensitivity during the game, which supports the emotional effects of the preceding results on their decisions. It can be inferred that average adolescents, regardless of their mathematical abilities, have more frequent interaction between affective and cognitive networks in the brain compared with the gifted. This interpretation of defective loss sensitivity in the gifted adolescents might account for the stereotypical portrayal of emotional maladaptation of gifted adolescents as compared to average adolescents [13], [14]. However, further research that focuses on neural activation differences and functional connectivity is required to compare with other controversial interpretations on interaction between brain regions [2], [7], [43].

Second, compared with gifted adolescents, defective gain sensitivity seemed to hinder average adolescents from taking risks. In the PG game, greed can be considered as a profit-maximizing ability that is related to gain sensitivity. As mentioned above, in condition II, participants had no risk of losing money, which eventually leads to greedy incentives for the participants. Hence, a player has a 50% chance of earning a bonus, and he or she can at least keep the endowment in the opposite cases. The decision process is still cognitively demanding, because the participants can maximize their profit by free-riding in about a third of the cases. Comparing the expected values for each option, it is a rational decision to cooperate in the corresponding condition. However, a greedy participant should free-ride to maximize total earnings. In our study, the average adolescents showed abnormally cooperative behavior during a condition without a risk of losing; they were relatively cooperative following trials in which they faced enough cooperators to earn a bonus (4 cooperators), but they failed to find an adequate strategy to maximize their profit during the game. It could be inferred from these results that mathematically gifted adolescents are more sensitive to the magnitude of the gain, and that this is represented as profit-maximizing behavior. Various previous studies suggested that asymmetrically developed right hemisphere might underlie the superior mathematical abilities of the gifted adolescents [11], [44]. The current result opens the possible neurodevelopmental differences between gifted and average adolescents might exist not only in the hemispheric dominance but also in the specific functional region, such as the prefrontal cortex, which is generally related to higher memory, cognitive performance, and reward process [45].

### Social cognition and social strategy in the PG game

During the PG game, we observed several different patterns between average and gifted adolescents in aspects of social decision-making. First, the gifted group succeeded more often than the average group in estimating others' next moves and in establishing cooperation rates high enough to earn a bonus. Average adolescents exhibited evidently abnormal behaviors that did not reflect the number of cooperators in the preceding trial. The ability to decide on a next move as an adequate reaction to others' decisions is associated with theory-of-mind (TOM) [46]–[49]. Recently, Moriguchi et al. [50] examined children and adolescents ranging from 9 to 16 and found that the activity of the neural substrates for TOM correlate significantly with age. It was implied that adolescence might be a critical period for maturation of the ability to process others' intentions in a complex social interaction. Thus, we speculate that gifted adolescents might be neuro-developmentally more mature than average adolescents in their ability to estimate others' intentions.

Another explanation for these behavioral differences is that average adolescents might have low levels of trust in their group and that they did not expect preserved high cooperation, as demonstrated by significantly decreased cooperation following trials with high rates of cooperation, when they did not benefit from free-riding (condition III). We also observed the effect of low trust in condition I, in which average adolescents cooperated significantly less following 3 cooperators than following 0 or 2 cooperators. We suggest that these distrusting behaviors, often observed in adolescents [51], might be strongly related not only to social cognition, but also to hypersensitivity to loss.

Additionally, average adolescents displayed herding behavior [52]. Especially in conditions I and II, average adolescents showed abnormally high cooperation in rounds following trials with 4 cooperators when compared with other cases (Fig. 4). Since these patterns appeared in the conditions that include greedy incentive, they could be considered as non-strategic but strong social adaptation within their age group. The current behavioral patterns support previous studies suggesting that a social affiliation dominated by peers powerfully motivates adolescents' decisions [53].

In aspects of strategic decision-making, average adolescents failed to find an optimal strategy that fit their group to maximize their own profit. Multiple factors such as loss sensitivity, herding behavior, and low trust in their group seemed to induce the results. In contrast to these social and emotional factors, the greed and risk-taking behavior that appeared in the gifted adolescents seemed to assist profit maximizing. A significant positive correlation in condition III between IQs and shift ratios of free-riders when they failed to earn a bonus additionally supports the idea that participants with higher IQs could manage and build group cooperation when their risk-aversive behaviors did not satisfy the bonus threshold. Strategic ability is known to be related to cognitive performance (e.g., working memory) and mathematical achievement [54], [55]. The current study demonstrates that mathematically gifted adolescents are superior in using economic strategy. However, at the same time, their strategic decision-making excludes social and emotional effects (e.g., herding behavior in the average adolescents); amongst average adolescents of their own age, this condition might cause social disharmony.

We found underpinnings of differences between average and gifted adolescents' behaviors concerning gain or loss sensitivity and social adaptation strategy during the PG game. Our findings must be interpreted in light of the limitations of this study. First, we assessed the relatively small number of subjects for each group. Thus, there were a few sub-cases that never occurred (e.g., the gifted group never had an instance of 4 preceding cooperators in condition I, and the average group never experienced 4 preceding cooperators in condition III), and some of the comparisons between groups or amongst sub-cases were restricted only to non-statistical and heuristic inspection. Second, the group size of the game in this study was set to five participants, which is relatively small compared with previous public goods studies (e.g., N = 4, 10, 40, 100) [17], [18]. Several studies showed that the level of cooperation is dependent on the group size [56]–[59]. Even though they also showed the increased cooperation in the large-group was not purely due to the members-in-the-group effect – *marginal per capita* return and critical mass is also related to the cooperativeness of the group, we should note that the behavioral characteristics in the current study might be limited to the current settings. Third, due to the complex decision-making processes required for the game, most of the underlying mechanisms are described qualitatively and concurrent recording of brain activity during the game was not possible, which will be our future investigation.

Nevertheless, the current study is the first report of the differential development of emotional and social or non-social cognitive abilities between average and gifted adolescents based on game theory. We estimated that, between the two groups, neuro-developmental differences in affective and cognitive crosstalk underlie the behavioral dissimilarity. Additionally, we suggest that uncovering gifted adolescents' low dependencies on social and emotional factors might pave the road for understanding the causes of their social isolation problem and provide more adequate educational systems for the gifted.

## Supporting Information

File S1 **Sequential effects on decisions in the PG game.**
(DOC)
