# Supplementary material for: Different Gain/Loss Sensitivity and Social Adaptation Ability in Gifted Adolescents during a Public Goods Game
Source: PLoS One. 2011 Feb 16;6(2):e17044. doi: 10.1371/journal.pone.0017044 (PMC3040203; doi:10.1371/journal.pone.0017044)
Supplement: File S1 — Sequential effects on decisions in the PG game. (DOC) [file pone.0017044.s001.doc]

# Sequential effects on decisions in the PG game

We analyzed cooperation changes over the course of repeated rounds in each condition. The gifted group showed degrading cooperation according to repeated trials (Fig. 1), which was similar to findings in adults in previous studies [1,2].


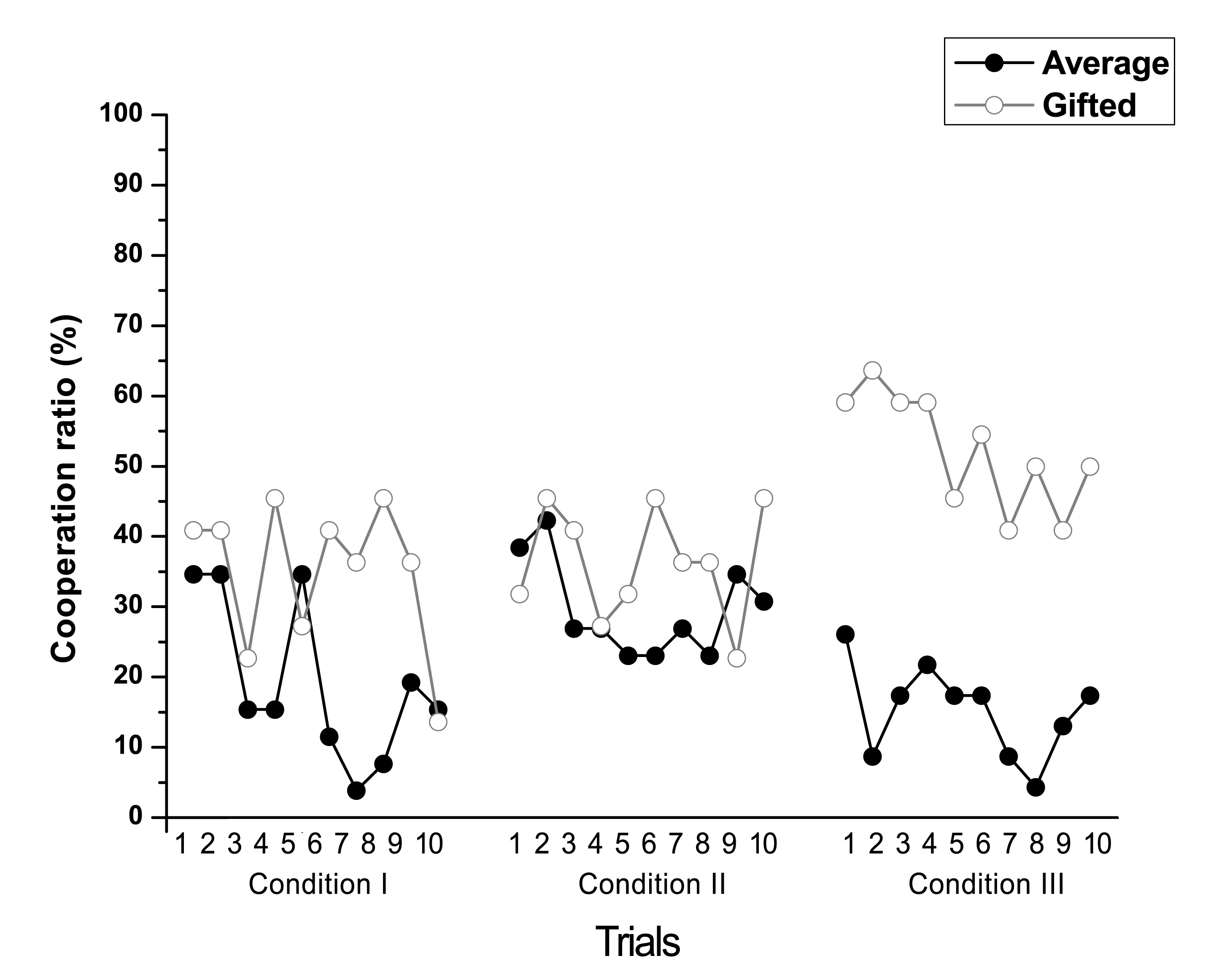


**Figure 1. Mean cooperation change according to the repeated trials in each condition.** The gifted adolescents showed decreasing cooperation through iterations in all conditions. The average adolescents exhibited similarly decreasing trends of cooperation in all conditions. In the regression analysis, we found a significant negative correlation of cooperation during the course of the iterations (slope: -2.01, p < 0.05). Chung, et al.

Cooperation decreased about 40% to 10% in condition I, 40% to 20% in condition II, and 60% to 40% in condition III. Regression analysis revealed insignificantly negative slopes in condition I (-1.07) and condition II (-0.11), but a significantly negative slope in condition III (-2.01, p < 0.05). In particular, decreased cooperation in later rounds of condition III indicates that gifted adolescents built little trust between group members during the game.

In the average group during condition I, we found drifts of about 20%, initially about 40%, from cooperators to free-riders. These individuals showed weakly decreasing cooperation rates: 40% to 30% in condition II, and 30% to 20% in condition III. In the regression analysis, we observed negative slopes in all three conditions (condition I: -2.28, condition II: -0.86, and condition III: -0.92), but the fits were not statistically significant. As we observed in the gifted group, the average adolescents also failed to build trust between group members.

Cooperation changes of the two groups were not statistically different in the regression analyses. However, behavioral differences between the groups were revealed by a 2-D color map (Fig. 2).


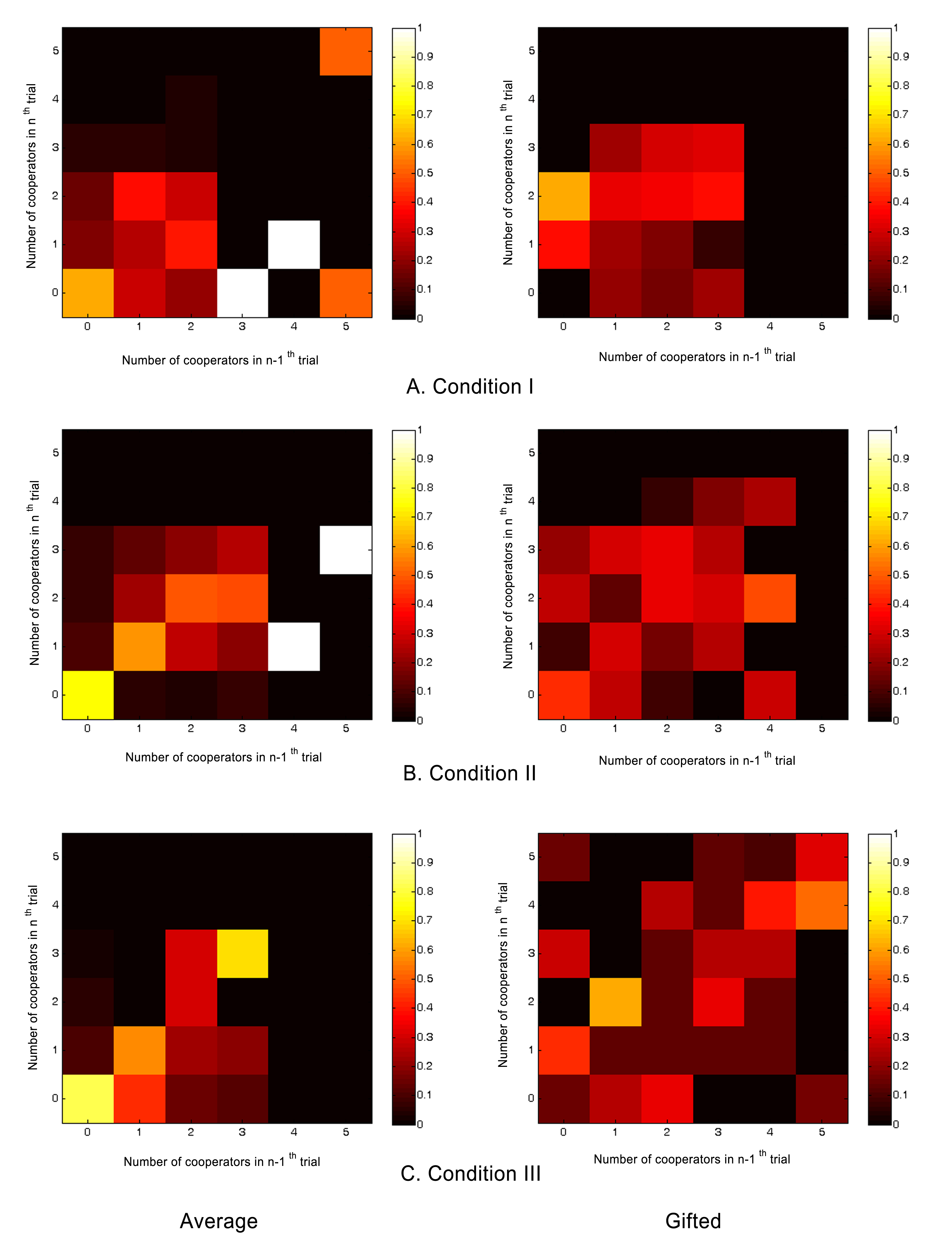


**Figure 2. Conditional probability of the number of cooperating participants in the next trial.** Each conditional probability was calculated given the number of cooperators in the preceding trial. The average students showed rather low and undifferentiated cooperation trends in all three conditions. The gifted adolescents exhibited a broader distribution of conditional probability over the number of presented cooperators in condition III than in conditions I and II, which displayed a rather localized low level of cooperation. Chung, et al.

We depicted the normalized frequency of the number of cooperators revealed in the successive round, matched to each case of cooperation in the preceding trial, i.e., the conditional probability of the number of cooperating participants in the next trial, given the number of cooperators in the preceding trial. The average adolescents showed little difference between the types of condition in which they participated. However, among the gifted adolescents, we observed a broader distribution of the conditional probability over the number of cooperators presented in condition III when compared with conditions I and II, which displayed a rather localized low level of cooperation.

# References

1. Camerer C (2003) Behavioral game theory: Experiments in strategic interaction: Princeton University Press Princeton, NJ.

2. Ledyard JO (1995) Public goods: A survey of experimental research. Handbook of Experimental Economics: 111-194.

**Table 1.** Significant correlations between the demographic data and the performance of the participants.

|  | | | IQ | ^a^KTCPI | | | | |
| --- | --- | --- | --- | --- | --- | --- | --- | --- |
|  |  |  |  | ^b^WKOPAY | | | ^c^SAM | |
|  |  |  |  | Self Confidence | Disciplined Imagination | | Initiative | Individuality |
| Cooperation | | |  |  |  | |  |  |
|  | | Condition II |  | **++(0.40)** |  | | **++(0.40)** | **+(0.32)** |
|  | | Condition III | **++(0.43)** |  | **++(0.44)** | |  | **+(0.30)** |
|  | | Succeed to condition II failure |  | **++(0.41)** |  | | **++(0.41)** | **+(0.32)** |
|  | | Succeed to condition III failure | **+(0.40)** |  | **++(0.42)** | |  |  |
| Total-earning | | |  |  | |  |  |  |
|  | Condition I | | **+(0.31)** |  | |  | **+(-0.35)** |  |
|  | Condition II | |  |  | |  |  | **+(0.36)** |
|  | Condition III | | **+++(0.52)** |  | | **++(0.45)** |  |  |

^a^KTCPI, Khatena-Torrance Creative Perception Inventory score; ^b^WKOPAY, What kind of person are you score; ^c^SAM, Something about myself score; Spearman’s correlation coefficient displayed in parentheses; +, p<0.05; ++, p<0.01; +++, p<0.001 (2-tailed).

**Table 1.** Significant correlations between the demographic data and the performance of the participants *(cont.)*.

|  | | ^a^KTCPI | | | | | |
| --- | --- | --- | --- | --- | --- | --- | --- |
|  |  | Average | ^b^WKOPAY | | ^c^SAM | | |
|  |  |  | Self Confidence | Inquisitiveness | Initiative | Intellectuality | Individuality |
| Stay ratio | |  |  |  |  |  |  |
|  | Condition I, cooperator, failure |  |  |  | **+(0.35)** |  |  |
|  | Condition I, free-rider, failure |  | **+(-0.29)** |  |  |  |  |
|  | Condition II, cooperator, success |  |  | **+(0.41)** |  |  |  |
|  | Condition II, cooperator, failure | **+(0.40)** |  |  |  | **+(0.44)** | **+(0.41)** |
|  | Condition II, free-rider, success |  | **+(-0.55)** |  |  |  |  |
|  | Condition II, free-rider, failure |  | **++(-0.42)** |  | **++(-0.46)** |  |  |

^a^KTCPI, Khatena-Torrance Creative Perception Inventory score; ^b^WKOPAY, What kind of person are you score; ^c^SAM, Something about myself score; Spearman’s correlation coefficient displayed in parentheses; +, p<0.05; ++, p<0.01; +++, p<0.001 (2-tailed).

**Table 1.** Significant correlations between the demographic data and the performance of the participants *(cont.)*.

|  | IQ | ^a^KTCPI |  | |
| --- | --- | --- | --- | --- |
|  |  | ^b^WKOPAY | |  |
|  |  | Self Confidence | | Disciplined Imagination |
| Stay ratio |  |  | |  |
| Condition III, free-rider, success |  | **++(-0.70)** | |  |
| Condition III, free-rider, failure | **+(-0.38)** |  | | **++(-0.48)** |

^a^KTCPI, Khatena-Torrance Creative Perception Inventory score; ^b^WKOPAY, What kind of person are you score; Spearman’s correlation coefficient displayed in parentheses; +, p<0.05; ++, p<0.01; +++, p<0.001 (2-tailed).
